# DoUBLing up: ubiquitin and ubiquitin-like proteases in genome stability

**DOI:** 10.1042/BCJ20230284

**Published:** 2024-04-04

**Authors:** Benjamin M. Foster, Zijuan Wang, Christine K. Schmidt

**Affiliations:** Manchester Cancer Research Centre (MCRC), Division of Cancer Sciences, School of Medical Sciences, Faculty of Biology, Medicine and Health, University of Manchester, 555 Wilmslow Road, Manchester M20 4GJ, U.K.

## Abstract

Maintaining stability of the genome requires dedicated DNA repair and signalling processes that are essential for the faithful duplication and propagation of chromosomes. These DNA damage response (DDR) mechanisms counteract the potentially mutagenic impact of daily genotoxic stresses from both exogenous and endogenous sources. Inherent to these DNA repair pathways is the activity of protein factors that instigate repair processes in response to DNA lesions. The regulation, coordination, and orchestration of these DDR factors is carried out, in a large part, by post-translational modifications, such as phosphorylation, ubiquitylation, and modification with ubiquitin-like proteins (UBLs). The importance of ubiquitylation and UBLylation with SUMO in DNA repair is well established, with the modified targets and downstream signalling consequences relatively well characterised. However, the role of dedicated erasers for ubiquitin and UBLs, known as deubiquitylases (DUBs) and ubiquitin-like proteases (ULPs) respectively, in genome stability is less well established, particularly for emerging UBLs such as ISG15 and UFM1. In this review, we provide an overview of the known regulatory roles and mechanisms of DUBs and ULPs involved in genome stability pathways. Expanding our understanding of the molecular agents and mechanisms underlying the removal of ubiquitin and UBL modifications will be fundamental for progressing our knowledge of the DDR and likely provide new therapeutic avenues for relevant human diseases, such as cancer.

## Introduction

The genome is constantly undergoing genotoxic stress due to endogenous processes, such as oxidation due to free radicals produced during cellular metabolism, or exogenous factors, such as ultraviolet (UV) light and ionising radiation (IR). In response, eukaryotic cells have evolved specialised mechanisms to detect and repair these DNA lesions to prevent their propagation to the next generation of cells in a process collectively known as the DNA damage response (DDR). Critically, the inability to detect or repair damaged DNA can lead to blocks in DNA replication or transcription, and mutation or chromosomal aberrations can occur leading to genome instability, a major hallmark of cancer [1]. Hereditary defects in the factors involved in these co-ordinated signalling pathways are associated with predisposition to immunodeficiency, neurodegeneration, infertility, premature aging, and most notably tumorigenesis, highlighting the importance of the DDR to human health [2,3].

The DDR enables cells to determine the type of DNA lesion and respond appropriately, such as by halting the cell cycle, altering gene transcription, instigating DNA repair, or to promote senescence or apoptosis if the damage is too severe. Importantly, post-translational modifications (PTMs) such as phosphorylation, ubiquitylation, and ubiquitin-like modifications play a critical role to enable a rapid and co-ordinated response to genomic damage and to switch off the response once genome integrity is reinstated. Phosphorylation brought about by phosphoinositide 3-kinase-like kinase (PIKK) family enzymes, ATM (ataxia-telangiectasia mutated), ATR (ataxia telangiectasia and Rad3-related protein) and DNA-PKcs, is well established to critically regulate DDR signalling pathways by activating specific repair factors and amplifying the cellular response in positive-feedback loops [4]. The factors involved and modified depend on the type of damage and the subsequent repair pathway. Translesion synthesis (TLS) allows replicative DNA polymerases to bypass blocking DNA lesions, while DNA interstrand cross-links (ICLs), caused by reactive mutagenic agents such as mitomycin C (MMC) or cisplatin, are repaired via the Fanconi anaemia (FA) pathway. The majority of IR-induced DNA double-strand breaks (DSBs) are repaired by homologous recombination (HR) or non-homologous end-joining (NHEJ), with HR being a higher fidelity repair pathway restricted to S/G2 phases of the cell cycle using a sister chromatid as a homologous template for repair. NHEJ, however, can often be error-prone but allows repair at any point in the cell cycle outside of mitosis. Other examples of repair pathways include base excision repair (BER) to correct non-helix-distorting base damage due to oxidising agents, nucleotide excision repair (NER) to respond to helix-distorting damage caused by UV light, and mismatch repair to revert base mismatches. Corresponding kinase activities generally relate to repair pathways, with ATM often linked to DSB repair, ATR associated with replication stress-coupled repair, and DNA-PKcs involved in NHEJ.

PTMs such as phosphorylation play numerous regulatory roles in cell signalling pathways in the DDR and beyond, such as inflammation and the cell cycle. Outside of alternative promoter usage and splicing, PTMs provide a key decision point to expand the human cellular proteome. While the role of phosphorylation has been long established in genome stability, ubiquitylation has been extensively studied with respect to proteasome-mediated degradation and in non-degradative roles (e.g. protein-protein interactions and protein localisation). Ubiquitin (Ub) signalling revolves around the addition of the small (8.5 kDa), highly conserved 76 amino acid long protein Ub to target substrates via an isopeptide bond between its C-terminal glycine to, predominantly, substrate lysine residues [5,6]. The covalent attachment occurs via an enzymatic cascade, with an initial ATP-dependent E1-activation step, followed by E2-conjugation, and E3-ligase mediated substrate modification. Ub modifications can exist as single (mono) forms, multi-mono (at multiple different substrate lysine residues) or as polymeric forms, with Ub polymers formed via attachment of Ub to the N-terminal methionine or one of seven internal lysine residues (K6, K11, K27, K29, K33, K48, and K63) within the Ub monomer [7]. The length and linkage type, whether as a homo-polymer, hetero-polymer, or as a branched chain, can dictate the downstream signalling consequences with alternative binding proteins, commonly referred to as readers or receptors, recruited depending on the poly-ubiquitin (polyUb) chain architecture [8–10]. Further complexity is introduced via the addition of PTMs on Ub, such as T12 [11,12] and S20, S57, and S65 phosphorylation [13], inclusion of ubiquitin-like (UBL) proteins, and non-lysine [14] or non-protein ubiquitylation [15,16]. Collectively, the resulting complex signalling repertoire is called the Ub/UBL code [8,17,18].

Other UBLs have been identified that differ in their primary amino acid sequence but share a common 3D structure with the ubiquitin molecule, specifically, a β-grasp fold and often a C-terminal glycine for covalent attachment to target substrates via enzymatic cascades analogous to the Ub system [19–21]. These include several paralogues of a small ubiquitin-like modifier (mainly SUMO1, SUMO2, and SUMO3) [22], neural precursor cell expressed and developmentally down-regulated 8 (NEDD8), interferon-stimulated gene 15 (ISG15), human leukocyte antigen F locus adjacent transcription 10 (FAT10), ubiquitin-fold modifier 1 (UFM1), ubiquitin-related modifier 1 (URM1), autophagy-related protein 12 (ATG12), autophagy-related protein 8 (ATG8), Finkel-Biskis-Reilly murine sarcoma virus (FBR-MuSV) ubiquitously expressed (FUBI), and ubiquitin-like protein 5 (UBL5). The involvement of Ub, SUMO, and NEDD8 in genome stability pathways has been well established [23–29], while the roles of ISG15 and UFM1 have only recently started to emerge as critical regulatory modules for the DDR [19,30].

**Figure 1.**
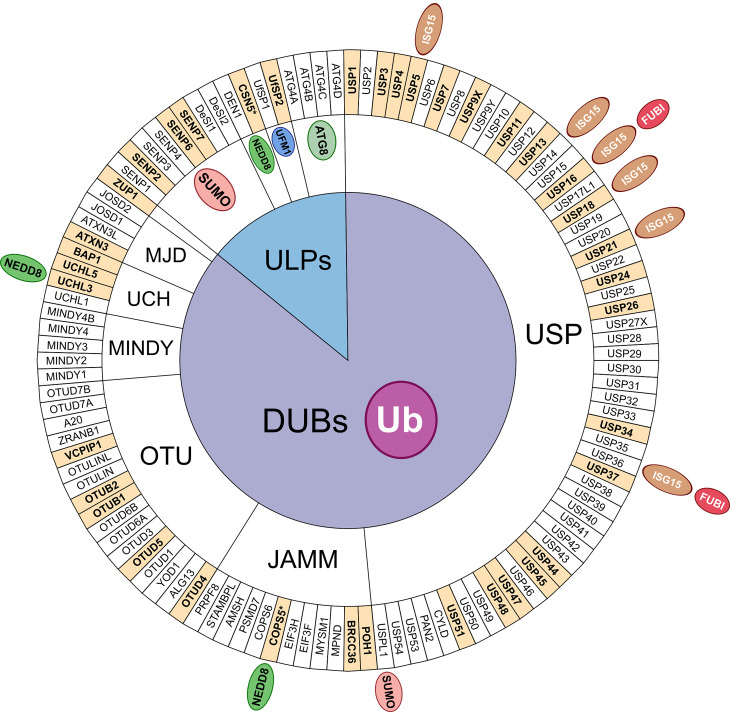
Circular plot of the human deubiquitylases (DUBs) and ubiquitin-like proteases (ULPs) currently known. Enzymes are grouped by major DUB families and proteases against UBLs. DUBs with additional ULP activity (e.g. USP18 and USP36) are annotated with the appropriate UBL outside of the circle. DUBs and ULPs referenced in this review to have a role in the DNA damage response (DDR) (Table 1) are highlighted in bold with a filled background. CSN5 and COPS5 (indicated by an *) are the same protein duplicated due to its annotation as a JAMM-family protease while having deNEDDylation activity. DEN1 is also known as NEDP1 or SENP8. ZUP1 is so far the only member of this DUB family and only a single member of USP17 (USP17L1) is shown due to its highly polymorphic copy number variation. ATG4A-D are cysteine proteases related to autophagy signalling pathways.

The identification and characterisation of the substrates, architecture, and readers for some of these UBLs is still in its infancy, but common to Ub and many of the UBLs is the existence of enzymes to cleave them at the terminal glycine residue, known as deubiquitylases (DUBs) for Ub and ubiquitin-like proteases (ULPs) for UBLs (Figure 1). DUBs and ULPs reverse the covalent attachment of Ub or UBLs from substrates or polymeric chains to remove the modification to counter proteolytic degradative pathways, alter the localisation or signalling output of the protein substrate, or in processing Ub/UBLs from their expressed precursor fusions. Therefore, the action of DUBs and ULPs has a key regulatory role within numerous cell signalling pathways, particularly with respect to genome stability [26,31–36]. Herein, we provide an overview of the current knowledge of DUBs and ULPs involved in diverse DDR signalling pathways, highlighting some of the mechanisms involved and future perspectives on identifying DUBs/ULPs in genome stability as potential therapeutic targets or markers for Ub/UBL-associated human diseases, such as cancer.

**Table 1.** Summary of key DUBs and ULPs involved in DDR signalling pathways.

DDR pathway	DUB or ULP (with references)	Substrates/function
DSB repair	BRCC36 [81–83,86,87,91]	K63 polyUb
	POH1 [89–91]	K63 polyUb
	USP5 [92]	Unanchored polyUb
	OTUB2 [93]	K63 polyUb
	USP26/USP37/USP44 [95,96]	RNF168-mediated Ub, H2A
	USP13 [97]	RAP80
	USP3/USP16/USP51 [94,98–101]	H2A/H2AX-Ub
	OTUB1 [102,103,105]	Non-catalytic E2 (Ubc13/UBE2N) inhibitor
	USP11 [106–111]	H2AK119/120-Ub; BRCA2/PALB2; RNF4-associated polyUb
	USP21 [112]	BRCA2
	UCHL3 [113]	RAD51
	BAP1 [114–116,121]	H2AK119, INO80 stabilisation
	USP48 [122]	H2AK125/127/129-Ub
	USP34/USP7 [123,124]	RNF168
	USP7 [127,128,133]	TIP60, MDC1, MRN, SUMO
	UCHL3/OTUD5 [148,149]	Ku80
	USP4 [150,151]	CtIP, MRN
	UCHL5 [80]	NFRKB
	SENP6 [242,252,254,255]	PolySUMO2/3
	SENP7 [254]	KAP1
	SENP2 [255]	MDC1
	ATXN3 [259,260]	MDC1, RNF8
	SENP6 [242,261]	BRCA1-BARD1, 53BP1, BLM, XPF-ERCC1, RPA
	UfSP2 [274]	H4, MRN
	CSN5 (CSN) [290,293,296]	CRLs, H4, CtIP
Replication stress	USP1 [165,166,174,175]	PCNA, FANCI-D2
	USP37 [198]	Cdt1, BLM
	VCPIP1 [195,196]	SPRTN
	USP7 [132–134]	SPRTN, SUMO
	ZUP1 [216–220]	K63 polyUb
	SENP6 [246]	FANCI-D2
	USP18 [30,280,281]	PCNA K164/168-ISG15
NER	USP7 [129–131]	CSB/ERCC6, XPC
	USP24/USP44 [224,225]	DDB2
	USP45 [226]	XPF-ERCC1
BER and DNA alkylation	USP47 [232]	Pol β
	OTUD4-USP9X-USP7 complex [235]	ALKB2/3

## Deubiquitylases

There are ∼100 DUBs encoded in the human genome that can be sub-divided into seven different classes: Ub C-terminal hydrolases (UCHs), Ub-specific proteases (USPs), Machado-Joseph domain-containing proteins (MJDs), otubain domain-containing proteases (OTUs), JAMM (JAB1/MPN/Mov34) metalloproteases, motif interacting with Ub (MIU)-containing novel DUB family (MINDY), and zinc finger-containing Ub-peptidase (ZUP1) [37,38] (Figure 1). Given the complexity of the Ub code, many DUBs display a certain level of selectivity for Ub chain architectures, such as preference for the predominantly proteasome-targeting K11- and K48-linked polyUb chains to protect substrates from degradation. Other DUBs, such as AMSH, BRCC36 and ZUP1, prefer cleavage of non-proteolytic K63-linked chains to alter binding or recruitment platforms in signalling pathways [37–41]. USP-family DUBs are generally promiscuous with respect to Ub chain linkage but are regulated via other means, such as through regulated gene expression, protein-protein interactions or specific recruitment/localisation, and PTMs such as phosphorylation or ubiquitylation [37,40,42–44]. DUB activity is required for the initial maturation of expressed Ub fusions to free Ub monomers from its precursors, predominantly by IsoT/USP5 [45,46], and the recycling of Ub following degradation of cellular proteins, by the DUBs USP14, UCHL5, and POH1 at the proteasome, with USP8 associated with ubiquitin homeostasis in lysosomal degradation pathways [47]. More generally, DUBs act to counteract the action of E3 ligases, which can modulate their signalling outcomes in different ways. For example, by removing proteasome-targeting Ub modifications they can protect proteins from proteolytic pathways. Alternatively, by selectively removing Ub moieties of a certain architecture they can edit the linkage type and topology of the polyUb chains present on the substrate protein. Therefore, DUBs are established to play key roles in several DDR signalling pathways with errors in their activity or function likely contributing to disease progression [31,35,48–50]. As a result, there is increasing appetite and potential for using small molecule inhibitors targeting DUBs in anti-cancer therapeutic strategies, with some already in early development [51–54].

### DUBs in DSB repair

An established role of Ub signalling is to recruit repair factors to sites of DNA damage or modulate their activity to co-ordinate the response. This means DUBs and their proper regulation are central to the maintenance of genome stability, particularly in the case of DNA DSBs [55]. DSBs are formed during endogenous processes such as DNA replication, or due to external factors such as IR, and can be repaired by HR during the S/G2 phases of the cell cycle or more error-prone NHEJ throughout interphase, independently of the cell cycle stage [56]. Classical NHEJ (c-NHEJ) relies on the heterodimer Ku70/Ku80 to bind DSB ends, which localises DNA-PKcs, XRCC4-LIG4, XLF, PAXX, plus other NHEJ factors to bring about synapsis of the DNA ends, DSB end-processing, and ligation of the break. HR, however, requires more extensive processing of the DNA that is initiated by the MRN (MRE11/RAD50/Nbs1) endonuclease complex to activate the ATM kinase and help recruit CtIP, BRCA1-BARD1, plus other helicases and nucleases, for long-range resection around the DSB. The resulting single-stranded DNA (ssDNA) is protected by the replication protein A (RPA) complex before BRCA2-mediated RAD51 loading, leading to strand invasion of the complimentary strand on the sister chromatid and subsequent repair. The cell cycle stage and the recruitment of either 53BP1 and RAP80 for NHEJ, or BRCA1-BARD1 for HR, are critical determinants for the method of repair.

**Figure 2.**
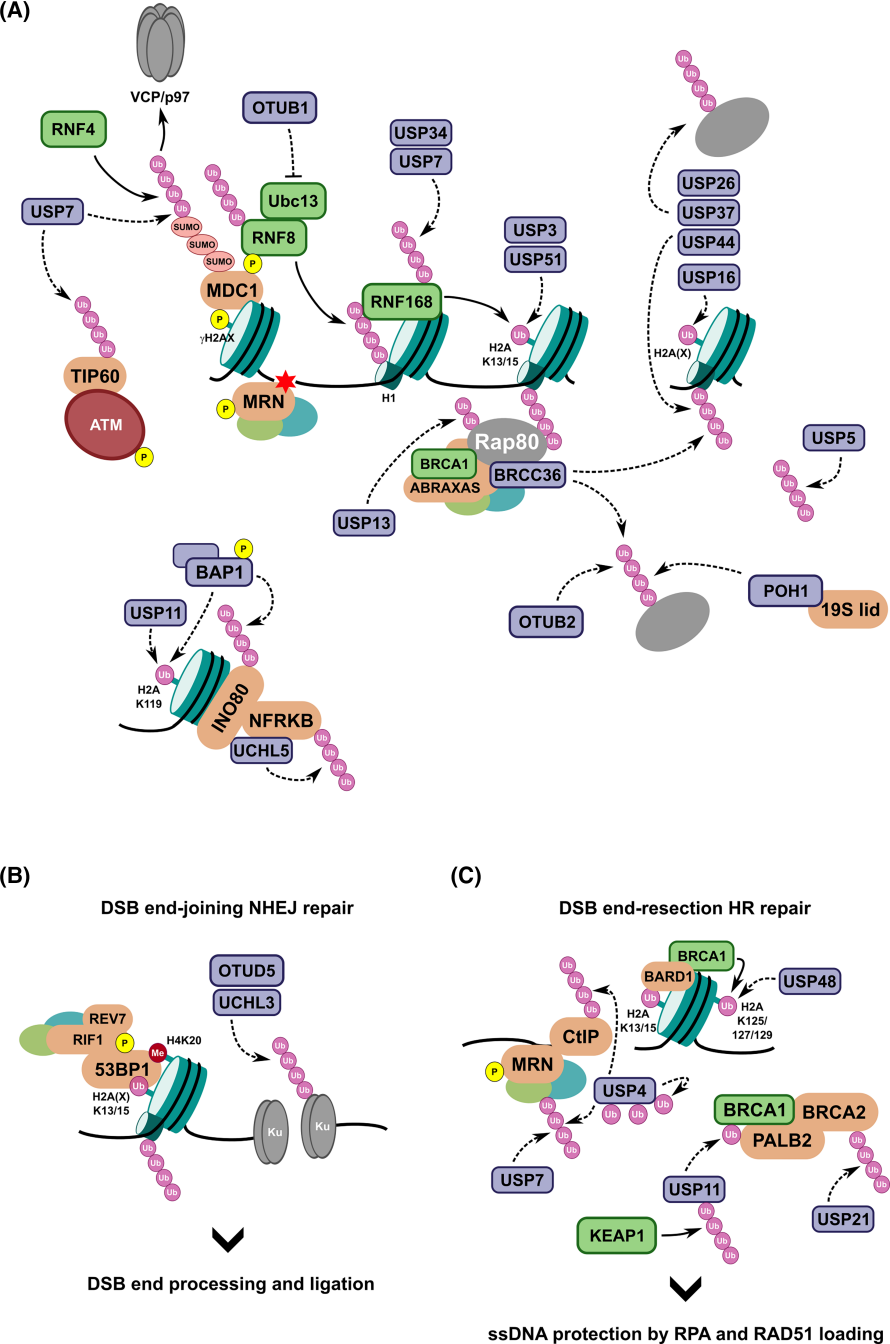
Overview of DUBs involved in DSB repair signalling pathways. (**A**) Upon DNA damage causing a DSB (red star), a plethora of signalling events involving Ub can occur to regulate the repair pathway choice in the context of other cell signals, such as cell cycle cues. MDC1 (SENP2 and USP7), TIP60 (USP7), RNF168 (USP34 and USP7), and RAP80 (USP13) are all stabilised through the action of DUBs to counteract polyUb-mediated protein degradation. RAP80 can bind both Ub and SUMO signals upon DSB formation to recruit BRCA1-A complex components, enabling sequestration of BRCA1-BARD1 and localisation of K63-linked polyUb DUB activity of BRCC36. Other DUBs, such as OTUB2, POH1, USP26, USP37, and USP44, dampen RNF168-mediated polyUb chain formation ensuring proficient repair of the DSB. USP51 can directly bind H2AK13/15-Ub-containing nucleosomes to remove the Ub signal, with USP3 and USP16 also implicated to remove H2A(X)-Ub modifications. USP11 and BAP1 can remove the polycomb-related H2AK119-Ub mark, likely to regulate transcription during DDR signalling and local chromatin accessibility, further aided by INO80 stabilisation through the DUBs BAP1 and UCHL5. (**B**) H2AK13/15-Ub combined with H4K20me2 is bound by 53BP1 in a multidentate manner to recruit the shieldin complex to help protect DNA ends and displace the HR repair machinery. Ku80 chromatin retention is regulated by the DUBs OTUD5 and UCHL3. (**C**) During S/G2 phase, the Ub E3 ligase BRCA1-BARD1 can bind H2AK13/15-Ub combined with unmethylated H4K20 to ubiquitylate H2AK125/127/129 to promote HR-mediated repair signalling. The DUB USP48 can counteract this Ub signal to prevent hyper-resection. The MRN complex and CtIP are stabilised at sites of DSBs through the action of USP4. The BRCA1-PALB2-BRCA2 complex, required for BRCA2-mediated loading of RAD51, is stabilised by cell cycle-regulated USP11, which removes an inhibitory Ub that otherwise disrupts the BRCA1-PALB2 interaction during S/G2 phase. DUB/ULP activity is represented by dashed arrows.

Ubiquitylation is central to the choice between HR and NHEJ for recruiting specific factors to the site of damage on chromatin (Figure 2A). Phosphorylation of the histone variant, H2AX, at S139 (forming γH2AX) by ATM kinase is a key initial signalling event [57,58] to recruit MDC1 [59], phosphorylation of which enables recruitment of the Ub E3 ligase RNF8 [60]. K63-linked polyUb chain formation on histone H1 by RNF8 [61–64], utilising the E2 conjugating enzyme Ubc13/UBE2N, leads to subsequent localisation of the Ub E3 ligase RNF168 to the damage site, resulting in further mono-ubiquitylation on histone H2A at K13/K15 (H2AK13/15-Ub) to propagate the signal [65]. RAP80 binding to K63-linked polyUb via a Ub-interacting motif (UIM) is thought to sequester the pro-HR Ub E3 ligase, BRCA1-BARD1, away from sites of DNA damage, while H2AK13/15-Ub in the context of dimethylated K20 on histone H4 enables 53BP1 recruitment [66–70]. 53BP1 accrual at DNA damage sites facilitates NHEJ by suppressing BRCA1 localisation and recruiting RIF1, REV7 and other components of the shieldin complex, which acts to protect DNA ends [71–73] (Figure 2B). The Ub E3 ligase RNF169 can also bind H2AK15-Ub [74–76], therefore competing with 53BP1 recruitment, to limit NHEJ signalling, and BRCA1-BARD1 ubiquitylation at H2AK125/127/129 can displace 53BP1 as an essential step to promote HR [77,78] (Figure 2C). Therefore, regulating target protein ubiquitylation and K63-linked polyUb chain formation is key and numerous DUBs have been identified to counteract aberrant Ub signalling to ensure robust DSB signalling, as detailed for key examples in the following section [31–33,48,79,80].

#### BRCC36 in the BRCA1-A complex

BRCC36 is a K63-specific metalloprotease of the JAMM-family of DUBs and was initially linked to the DDR through localisation studies indicating its presence at nuclear foci for DSBs [81,82]. This recruitment was dependent on RAP80 as part of the BRCA1-A complex, which also contains BRCA1, BARD1, MERIT40, BRCC45 and Abraxas, and plays an important role to sequester BRCA1 away from DSB sites and preventing end-resection pathways [83–85] (Figure 2A). Recent structural data shows how the BRCA1-A complex, different from the cytoplasmic BRISC complex that also contains BRCC36, can engage and sequester BRCA1 while enabling RAP80 to bind mixed Ub-SUMO linked chains, relevant for its role in regulating DSB repair outcomes [86]. Depletion of BRCC36 results in elevated K63-linked polyUb at DSBs, leading to increased 53BP1 foci formation, but cells exhibit a hyper-HR phenotype with increased resection, RAD51 loading, and IR sensitivity, suggesting loss of BRCC36 leads to unproductive repair [87,88]. Therefore, preventing excessive K63-polyUb chain formation at DSBs seems to balance the signals alongside other histone PTMs and cell cycle stage, enabling conditions conducive to both HR and NHEJ.

#### POH1 linking DDR signalling to the proteasome

POH1, otherwise known as Rpn11 or PSMD14, is a proteasome-associated DUB that has been linked to the DDR [89,90]. Like BRCC36, POH1 is a JAMM-family metalloprotease able to cleave K63-linked polyUb chains, as well as other chain types [90,91]. Depletion of POH1 results in increased sensitivity to DSBs in response to hydroxyurea (HU) with elevated polyUb foci at damaged sites. POH1 DUB activity against K63-linked polyUb restricts 53BP1 accumulation at DSB foci, antagonising RNF8/RNF168, and therefore modulating NHEJ-mediated DNA repair and promoting HR-mediated resection pathways [90]. POH1 also acts independently of 53BP1 to promote RAD51 loading in HR-mediated repair during G2 phase of the cell cycle, meaning POH1 is a key component of DSB repair pathways to limit anchored polyUb conjugates at sites of damage to ensure proficient repair [89]. The activity of POH1/Rpn11 to bulk-cleave polyUb chains from substrates, such as histone proteins to counteract 53BP1 binding, may be linked to another DUB USP5, which has known DUB activity against unanchored polyUb chains [46], to recycle the Ub protein for signalling elsewhere. Depletion of USP5 also results in DNA repair defects and USP5 is thought to be recruited to sites of DNA damage [80,92].

#### OTUB2 as an antagonist to RNF8/168 signalling

An RNAi screen identified another K63-specific DUB, a member of the OTU-family of DUBs OTUB2, which antagonises RNF8/RNF168-mediated Ub signalling in the DDR [93]. Depletion leads to increased Ub, 53BP1, RAP80, and RNF168 foci at damage sites, resulting in increased preference for NHEJ-mediated repair pathways, while overexpression does not alter recruitment of other upstream factors such as RNF8 and MDC1. Therefore, OTUB2 likely supresses K63-linked polyUb chain formation to prevent RNF168, and therefore 53BP1, recruitment and favouring HR-mediated repair. Despite both being K63-specific DUBs, depletion of BRCC36 and OTUB2 have opposing outcomes, with BRCC36 depletion leading to a hyper-resection phenotype. Therefore, differing DUB actions, such as substrate choice and site of action, may influence the recruitment and coordination of the HR-suppressive RNF168-RAP80-53BP1 components, or they may have additional functions in repair outside of their intrinsic DUB activity (e.g. BRCC36 as part of the BRCA1-A complex) resulting in alternative outcomes. Additional DUBs that limit or counteract RNF168-mediated ubiquitylation include USP16, which is thought to regulate DSB signalling through H2A(X) deubiquitylation [94], and USP26, USP37 [95], and USP44 [96] that have been identified as DUBs recruited to sites of DSBs to counteract RNF168-mediated ubiquitylation. USP13, on the other hand, reverses an inhibitory ubiquitylation on RAP80 close to its UIM, which disrupts its binding to K63-linked polyUb, enabling BRCA1-A recruitment to DSBs [97].

#### DUBs linked to histone H2AK13/15-Ub

In addition to the polyUb DUB activity described above, several DUBs have been identified to act directly on the H2AK13/15-Ub mark. USP3 [98,99], and USP51 [100] have been described to have such activity and counteract RNF168-mediated action on H2A and/or H2AX, so suppressing DSB repair signalling. In the case of USP51, it has been shown to be recruited to chromatin and directly bind the site of action on the H2A/H2B dimer upon DNA damage to specifically remove Ub from H2AK13/15 [101], with knockdown resulting in increased spontaneous DNA damage and elevated 53BP1 and BRCA1 foci formation due to increased H2AK13/15-Ub, while not affecting upstream signalling events.

A common theme is the apparent redundancy between DUBs in the DDR, such as for USP3, USP16, and USP51 noted above for H2A ubiquitylation, with likely effects on how the response to targeted drugs impacts on the signalling outcome. Whether some of this redundancy is due to cell-type differences in expression, lack of specificity (common for many DUBs *in vitro*), or if they have experimental explanations would need to be determined. Another possibility is that the non-catalytic roles of some DUBs may have additional effects on the DDR signal. One such example is OTUB1 [102]. OTUB1 is a negative regulator of RNF168-mediated polyUb chain formation at sites of DNA damage [103]. OTUB1 inhibits the E2 enzyme, Ubc13/UBE2N plus other E2 conjugating enzymes, independently of its DUB activity by binding directly to the charged E2∼Ub via an N-terminal extension that contains a Ub-binding motif [104,105]. This prevents the transfer of Ub from the E2 to the target substrate. OTUB1 depletion results in spontaneous and persistent polyUb and 53BP1 foci, and therefore regulates DSB repair pathway choice.

#### USP11 in DSB repair pathway choice

USP11 is another DUB, identified in an overexpression screen with ectopic RNF8, to act on ubiquitylated H2A [106,107]. Validation with USP11 depletion showed increased H2AX-Ub, increased 53BP1 and polyUb foci, and increased sensitivity to IR suggesting improper DSB repair signalling. USP11 can physically interact with H2AX and localises to sites of DNA damage, reinforcing its role in DSB repair signalling, while its activity against H2A-Ub is proposed to be at K119/K120 in H2A, rather than the RNF168/53BP1-relevant H2AK13/15 [107] (Figure 2A). USP11 has also, however, been implicated as an interacting partner and DUB for various other factors in the DDR, particularly in DSB repair, making its mode of action much more complex. USP11 is regulated in a cell cycle-dependent manner by KEAP1 [108], with USP11 levels being low during G1. Indeed, USP11 has been shown to bind and remove Ub from PALB2 to facilitate the formation of the BRCA1-PALB2-BRCA2 complex enabling RAD51 loading and HR repair [108,109], with USP11 abundance during S/G2 able to facilitate this (Figure 2C). It has also been identified as an interactor of the SUMO-targeted Ub ligase (STUbL) RNF4 to counteract its ubiquitylation activity, preventing SUMO-Ub hybrid chains from forming, and regulating DSB repair signalling choice [110]. Consistent with these roles in HR-mediated DSB repair, USP11 depletion sensitises cells to olaparib (PARPi) and IR [111]. Additionally, USP21 was identified to stabilise the BRCA2-RAD51 complex by antagonising BRCA2 ubiquitylation [112], while UCHL3 counteracts RAD51 ubiquitylation [113], both ensuring competent RAD51 loading and proficient HR repair.

#### BAP1 stabilises the INO80 remodelling complex

**Figure 3.**
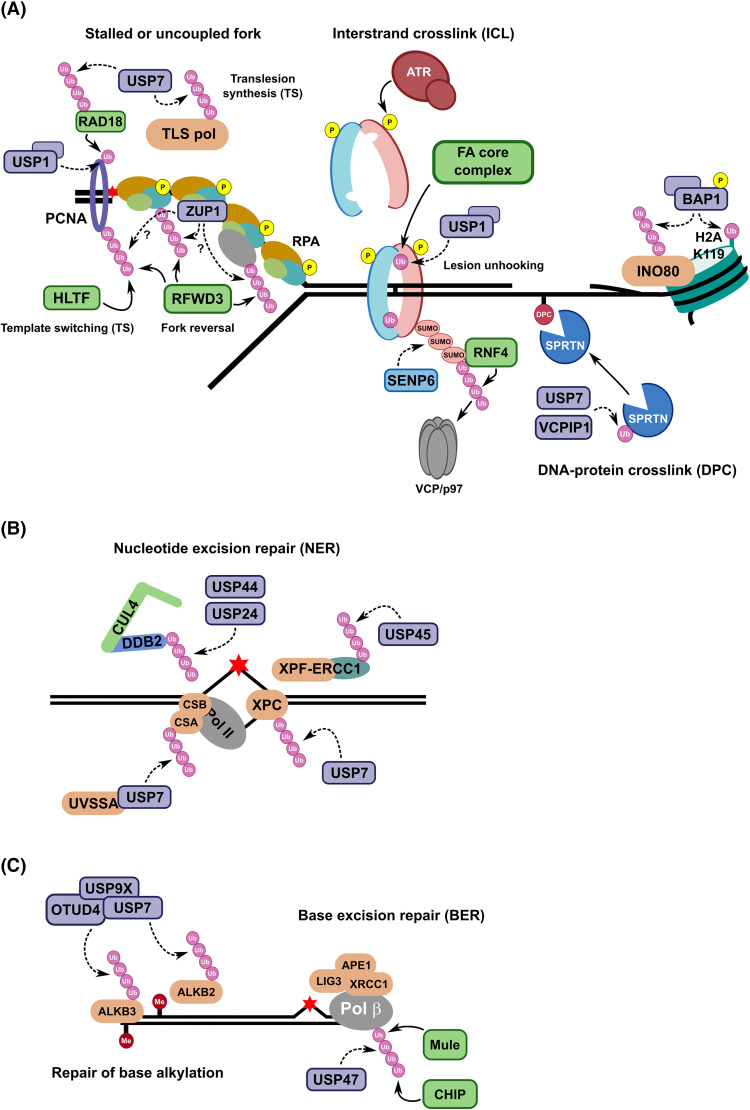
Overview of DUBs in DDR signalling pathways associated with replication stress, NER, and BER. (**A**) DUBs act on central signalling nodes within the DDR signalling pathways upon replication stress. PCNA is mono-ubiquitylated by RAD6/RAD18 upon stalling or slowing of the replication fork due to DNA damage. Mono-ubiquitylated PCNA forms a platform to recruit more error-prone TLS polymerases to bypass the lesion. RAD18 and the TLS polymerases are themselves protected from polyUb-mediated degradation via the proteasome due to the action of USP7. To restore normal replicative processes, the mono-Ub is removed by USP1-UAF1. Template switching (TS) is thought to be instigated by HLTF- and SHPRH-dependent poly-ubiquitylation of PCNA, recruiting the helicase and endonuclease ZRANB3 as the downstream effector. Replication stalling can lead to excessive ssDNA bound by ATR phosphorylated RPA, which can lead to replication fork reversal or TLS via RFWD3-dependent poly-ubiquitylation of surrounding proteins, including of PCNA and RPA. ZUP1 is hypothesised to be a candidate DUB to counteract polyUb chain formation during this process. Interstrand cross-links (ICLs) are generally repaired via the Fanconi anaemia (FA) repair pathway. FANCI-D2 is ubiquitylated by the FA core complex in an ATR-dependent manner, resulting in the FANCI-D2 heterodimer forming a DNA clamp at the site of damage to facilitate downstream repair. USP1-UAF1 can remove the mono-Ub mark. SENP6 is a deSUMOylase that counteracts polySUMO2/3 chain formation on FANCI-D2, preventing RNF4-mediated poly-ubiquitylation leading to VCP/p97-mediated extraction of FANCI-D2 from chromatin. One mechanism to remove DNA-protein cross-links (DPCs) is through the DNA-dependent metalloprotease SPRTN whose activity requires removal of an inhibitory mono-Ub by USP7 and/or VCPIP1. BAP1 stabilises the chromatin remodeller INO80 through its DUB activity to provide a DNA repair-proficient chromatin environment upon damage. (**B**) DUBs in nucleotide excision repair (NER) primarily protect repair factors from degradation. CUL4-DDB2 forms an E3 ligase for the process, with USP24 and USP44 thought to stabilise DDB2 by removing polyUb chains. One of the substrates for CUL4-DDB2 is XPC, which is protected from chromatin extraction and degradation by the action of USP7, with USP7 also removing polyUb from CSA via UVSSA-mediated recruitment. The endonuclease, XPF-ERCC1, is retained at sites of damage by USP45. (**C**) During base excision repair (BER), Pol β abundance and chromatin engagement is regulated through Ub signalling by the E3 ligases Mule and CHIP, with the DUB USP47 able to remove the polyUb signal. Repair of DNA base alkylation by ALKB2 and ALKB3 is enabled by a DUB complex made up of USP9X and USP7, with OTUD4 forming a non-catalytic scaffold subunit. DUB/ULP activity is represented by dashed arrows.

BRCA1-associated protein 1 (BAP1) is a nuclear-localised UCH-family DUB originally reported as a tumour suppressor, with its DUB activity linked to promoting HR-mediated repair of DSBs [114]. Depletion of BAP1 leads to reduced BRCA1 foci, and reduced RAD51 loading, with reduced HR repair and increased IR sensitivity coinciding with elevated H2A and H2AX ubiquitylation. The target for BAP1 is H2AK119, a PRC1-related histone ubiquitylation, potentiating a role for BAP1 in the transcriptional or chromatin response to DNA damage, rather than a direct role in the repair process [115]. Linked to this is the role of BAP1 in replication-coupled repair, where BAP1 aids to recruit and stabilise the chromatin remodeller INO80 to sites of DNA damage or replication stress, possibly to promote resection through its remodelling activity [116] (Figures 2A and 3A). BAP1 recruitment to sites of DNA damage is rapid and dependent on PARP1 and/or ATM-mediated modification of BAP1, with it also having a role in NER signalling pathways via PARP1 [54,117,118]. Recent structures of BAP1/ASXL1 with H2AK119-Ub-containing nucleosomes highlighted the role of many BAP1 mutations identified in cancer, likely to have roles in transcriptional regulation or DNA repair pathways linked to its role as the polycomb-repressive DUB (PR-DUB) [119–121].

#### USP48 counters hyper-resection

BRCA1-BARD1 mediated H2A ubiquitylation at K125/127/129 also needs to be regulated to dampen HR and end-resection repair [77,78]. USP48 has been identified to reverse this modification by specifically removing Ub at this site [122] (Figure 2C). Depletion of USP48 results in hyper-resection leading to single-strand annealing (SSA) as a method of repair, which is highly mutagenic due to the use of homologous repeats for bridging DSBs leading to genomic deletions.

Other DUBs have been identified to regulate the HR/NHEJ pathway choice through directly regulating RNF168, rather than the downstream H2A/H2AX ubiquitylation. USP34 [123] and USP7 [124] directly associate with RNF168 to prevent its poly-ubiquitylation and subsequent degradation via the proteasome (Figure 2A). Therefore, their role is to promote Ub conjugation at DSBs to enable downstream recruitment of 53BP1 and BRCA1, conferring resistance to DNA damaging agents. However, levels need to be regulated to prevent improper spreading to undamaged chromatin and two HECT-family ligases, UBR5 and TRIP12, have been shown to negatively regulate RNF168 abundance and spreading at DSBs [125]. While an alternative mechanism to DUBs described above, a common paradigm in DSB repair signalling is regulated RNF168 recruitment, and subsequent deposition of H2A-Ub marks, to enable proficient repair in the context of other signals such as cell cycle cues.

#### Multifaceted roles of USP7 in DDR signalling

The role of USP7 in genome stability is reviewed more thoroughly elsewhere [126], given its vast number of substrates identified and roles in a multitude of cell signalling pathways. In the context of DSBs, it stabilises the acetyltransferase TIP60 that acetylates ATM to promote and propagate DNA damage signalling [127], as well as binding to, and stabilising, MDC1 and [128] (Figure 2A). USP7 is involved in NER in complex with UVSSA to regulate CSB/ERCC6 stability [129,130] or as a DUB for XPC to promote its chromatin loading and stability [131] (Figure 3B). USP7 has also been implicated in the protection against replication stress as a DUB for removing Ub from SUMO chains to protect RAD18 and Pol Eta (η) from proteasome-mediated degradation [132,133], and to remove the inhibitory Ub modification on the DNA-dependent metalloprotease involved in removing DNA-protein cross-links (DPCs), SPRTN [134] (Figure 3A). While not explored in detail here, USP7, along with its role in p53 signalling [135], is critical for genome stability and disease progression, and is actively being investigated from a therapeutic perspective [136–142], with structural characterisation of highly selective USP7 inhibition well established [143–147].

Several other DUBs have been identified to play a role in regulating components of DSB repair UCHL3 [148] and OTUD5 [149] have both been postulated to counteract Ku80 ubiquitylation to stabilise chromatin retention of the Ku heterodimer to promote NHEJ (Figure 2B). USP4 has been linked to positively regulate DNA end resection. USP4 interacts with CtIP, in addition to NBS1 as part of the MRN complex, with USP4 depletion reducing the recruitment of CtIP, RPA, and RAD51 loading to DSB sites [150]. The interaction relies on auto-deubiquitylation of USP4, as a catalytic mutant, unable to remove the polyUb chains on itself, could not interact with CtIP or be recruited to sites of DSBs [151]. UCHL5/UCH37 was identified in a siRNA screen to promote end resection and HR-mediated repair of DSBs [80]. UCHL5 is associated with both the proteasome lid and the INO80 chromatin remodelling complex. With respect to genome stability, the role of UCHL5 is to remove polyUb chains, and subsequent degradation, of NFRKB (nuclear factor related to kB-binding protein), a factor known to promote the interaction between UCHL5 and INO80 (Figure 2A). As noted above, INO80 is required to promote end resection and overall DSB repair.

### DUBs in DNA replication-coupled repair

DUB involvement in HR and NHEJ pathways enables proper regulation of the DSB repair choice, dependent on other PTMs, proteins, and cell cycle cues that influence proficient DNA repair. During DNA replication, several repair pathways converge (e.g. HR; SSA; TLS; ICL repair; template switching, TS) because of replication stress to prevent fork collapse and replication catastrophe [152–154]. The type of damage is thought to recruit specific DDR factors to ensure competent repair. Central to the response to replication stress, DPCs, and ICLs is Ub modification of proteins at the fork [155–157], and a few key DUBs have been identified to enable tight regulation of these processes (Figure 3A).

#### USP1 in DNA replication-coupled repair pathways

USP1 is a DUB that is established to play a key role in the protection against ICL damage and replication stress. A central node for Ub signalling in replication stress is PCNA (proliferating cell nuclear antigen), which functions as the sliding clamp for replicative polymerases ensuring processivity during DNA synthesis. PCNA can be modified with Ub at the conserved K164 position in response to DNA damaging agents (e.g. HU, aphidicolin, UV), resulting in the slowing or stalling of replication forks [158–160]. The mono-ubiquitylation is brought about by RAD6 (E2) and RAD18 (E3) and leads to the recruitment of lower fidelity Y-family TLS polymerases such as Rev1, Pol η (eta), κ (kappa), and ι (iota) via their Ub binding domains (UBDs) and weak PCNA interacting peptide (PIP) box interactions, that allow for bypass of DNA lesions during replication [161–164]. Once the damage is passed or repaired, the PCNA-Ub mark needs to be reversed to resume high-fidelity DNA replication to maintain genome integrity and prevent error-prone replication. This activity is performed by USP1, in complex with UAF1 (also known as WDR48), with dysregulation of USP1 activity leading to increased PCNA ubiquitylation and mutational frequency, reduced fork speed, and micronuclei formation [165–167]. The recruitment of USP1-UAF1 is thought to be via a SUMO-like domain on UAF1 that recognises the SUMO-interacting motif (SIM) on ELG1, a PCNA-binding RFC-like adaptor for Ub removal from PCNA [168,169]. USP1 is also regulated by a self-cleavage mechanism occurring at an internal GG motif at residues 670–671, leading to rapid degradation of USP1 in response to UV-induced DNA damage [165]. Interestingly, a recent study has identified USP1 autocleavage-defective mutants, similar to a catalytic mutant, that lead to increased retention at sites of DNA synthesis leading to the accumulation of USP1 trapping lesions on DNA, which can partially be alleviated by SPRTN to ensure genome stability [170,171]. USP1 is a topical target for cancer therapeutics [172], particularly given its synthetic lethality with BRCA1 [167], and it is possible that pharmacological inhibition of USP1 may lead to USP1 trapping in addition to abrogation of its DUB activity, and therefore may explain some of the effects seen. A recent study highlights how ML323 may engage with USP1, providing a platform for subsequent drug development for its inhibition [173]. Another small molecule, KSQ-4279, has been identified in CRISPR screens and has entered Phase I clinical trials as a monotherapy or combination therapy with PARP inhibitors or chemotherapy [53], highlighting the promise of therapeutically targeting DUBs, such as USP1.

USP1-UAF1 also contributes to genome stability as a DUB for FA repair of ICLs caused by MMC or cisplatin [166,174,175]. ICLs can block DNA replication and transcription if left unrepaired, with FA signalling coordinating multiple repair pathways including NER, HR, and TLS depending on the specific type of damage. A key step in FA signalling is the Ub modification of the FANCI and FANCD2 (FANCI-D2) heterodimer at residues K523 and K561, respectively, by the FA core complex [165,176–178]. Mono-ubiquitylation of FANCI-D2 is activated by ATR-mediated phosphorylation and peaks during S phase of the cell cycle or in response to ICL-inducing agents [179–182]. Recent structural data indicates that sequential ubiquitylation results in substantial conformational changes, enabling ubiquitylated FANCI-D2 to form a clamp around the double-stranded DNA (dsDNA). This likely protects the underlying DNA during the DDR [183–190] and facilitates downstream repair. Interestingly, the mono-Ub mark does not, to date, seem to mediate the recruitment of repair factors, rather the Ub marks are bound by the other member of the heterodimer in FANCI-D2 effectively shielding it from UBDs in FA factors. For competent repair, however, the Ub modification needs to be removed from FANCI-D2 to facilitate its removal from chromatin [166,174,175]. USP1 was identified in an RNAi screen, as depletion increases spontaneous FANCD2 ubiquitylation and increases cellular sensitivity to ICL-inducing cross-linking agents. Structural data of USP1-UAF1, with Ub and in an enzyme-substrate complex with ubiquitylated FANCI-D2, reveals critical mechanistic and regulatory details for Ub removal from FANCD2 [191–194]. Amino acid residues at the FANCI-UAF1 interface, including those of known ATR phosphorylation sites, are crucial for USP1-mediated removal of the FANCD2-Ub mark, corroborating genetic and biochemical data.

#### Other DUBs involved in countering replication stress

Other DUBs have been proposed to have roles in other aspects of replication-coupled repair pathways. USP37 has been suggested to stabilise the DNA replication origin licensing factor, Cdt1, to ensure proficient DNA replication and to protect the RecQ helicase, BLM, from proteolysis [195–197]. DPC repair via the DNA-dependent metalloprotease SPRTN is regulated through an inhibitory Ub modification, removal of which is thought to be via USP7 [134] and VCPIP1 [198]. VCPIP1 is activated and shuttled to the nucleus by ATM/ATR-mediated phosphorylation, where it can remove the Ub mark, enabling SPRTN recruitment to chromatin and downstream repair of the DPC.

#### Removing polyUb signals at sites of replication stress

Ub signalling in replication-coupled repair and its regulation by DUBs has predominantly focussed on mono-Ub modifications on DDR factors. PolyUb chains have been identified to occur on PCNA [199,200]; RPA [201–205]; and the CMG (Cdc45-MCM-GINS) complex [206–210]. TRAIP-dependent polyUb chain formation on the CMG helicase is proposed to be linked to VCP/p97 extraction upon replication termination or helicase stalling for unloading [209,211,212]. The role of PCNA poly-ubiquitylation by Rad5 in yeast, HLTF is the human homologue, has been linked to TS through association with the helicase and endonuclease, ZRANB3, and another Ub E3 ligase, SHPRH, to promote error-free fork restart [199,213]. Similarly, RPA poly-ubiquitylation is non-degradative to promote DNA damage bypass and fork restart (Figure 3A). DUBs for these processes have not been robustly elucidated, with the polyUb signal likely leading to VCP/p97-mediated extraction [214]. A recently discovered DUB with preference for K63-linked polyUb chains, ZUP1, has been associated with replication-coupled genome stability [215–219]. ZUP1 localises to sites of DNA damage, interacts with the RPA complex, and depletion increases spontaneous DNA damage and sensitises cells to DNA damaging agents such as camptothecin (CPT). This activity relies on the DUB activity of ZUP1, and preliminary data suggested it to be the DUB for ubiquitylated RPA. A recent study further proposed that the direct interaction with the RPA complex enhanced the K63-linked polyUb DUB activity of ZUP1, suggesting that ZUP1 is recruited and activated through binding to the critical ssDNA binding protein complex [220]. The identity of polyUb modified substrates for ZUP1 at sites of DNA damage remain unknown, but one hypothesis is that it can counteract ssDNA-associated K63-linked polyUb chain formation from E3 ligases, such as RFWD3.

### DUBs in the repair of DNA adducts

The repair of bulky DNA adducts, such as thymidine dimers induced by UV light, is carried out by NER, reviewed in more detail elsewhere [221–223]. Recognition of the damage can occur via transcription-coupled NER (TC-NER) or by global genome NER (GG-NER), before the pathways converge to correct the damage. XPC and DDB proteins act as global genomic DNA damage sensing factors to recognise the lesion and recruit the repair machinery. At transcriptionally active regions, TC-NER relies on stalling of the RNA polymerase, with associated CSA and CSB proteins enabling damage recognition without the need for global distortion surveillance. Dual incision of the damaged site occurs via XPG and XPF-ERCC1 endonucleases before DNA polymerase loading to fill-in the damaged strand and sealing by a DNA ligase.

Ub signalling in NER, and its regulation by DUBs, revolves around the action of USP7 on XPC and CSA, mentioned above (Figure 3B). Another DUB, USP24, is also required for proficient repair and cell survival [224]. USP24 has been found to bind, deubiquitylate, and stabilise DDB2, a component of the CUL4 E3 ligase. CUL4-DDB2 is known to poly-ubiquitylate XPC, a substrate of USP7, so how these DUB activities act in concert to regulate NER is unclear. USP44 is another DUB that has been implicated to stabilise DDB2 and shown to prevent tumour progression in mice [225]. The DNA repair endonuclease XPF-ERCC1 is regulated through the DUB USP45 in response to UV damage. USP45 associates with ERCC1 and depletion results in increased levels of ubiquitylated ERCC1, leading to hypersensitivity to UV irradiation [226]. In cells, USP45 knockout, rather than leading to increased ERCC1 proteolysis, causes aberrant ERCC1 translocation to DNA damage foci, likely accounting for defective DNA repair.

BER is an essential repair pathway to remove lesions due to deamination, oxidation, and alkylation of DNA caused by IR or reactive oxygen species [227,228]. The removal of the damaged base by specific glycosylases leads to formation of an abasic site (AP site) that is cleaved by an AP endonuclease (APE1) resulting in a single-strand break, which is repaired by a complex including DNA polymerase β (beta), XRCC1, and DNA ligase III that are recruited in a PARP1-dependent manner. This process is known as single nucleotide or short-patch BER, while long-patch BER relies on the activity of RECQ1, RPA, XPF-ERCC1, and FEN1, which can remove the 5'-flap structure in a PCNA-dependent manner before filling in by polymerase and DNA Ligase I [229–231]. Pol β forms a central node to the BER process, coupling AP lyase and polymerase activities, and levels are tightly regulated to prevent increased mutagenesis and cancer susceptibility. Pol β levels are normally in proportion to the level of damage, with excess pol β targeted for proteasome-mediated degradation through sequential mono-ubiquitylation then polyUb chain formation by the E3 ligases Mule and CHIP, respectively. USP47 has been identified as the DUB counteracting this process to maintain proper levels of pol β, with depletion resulting in reduced levels and deficient BER [232] (Figure 3C).

#### A DUB complex involved in repair of DNA alkylation

Alkylation of DNA is a frequent mutagenic event caused by endogenous agents, such as *S*-adenosyl methionine, or external chemicals such as methyl methanesulfonate. In humans, ALKB2 and ALKB3 are the prominent oxidative demethylases involved to remove the modification, with ALKB2 preferring methylated dsDNA as a substrate and ALKB3 for ssDNA or RNA [233]. The stability of these enzymes is regulated by Ub signalling and the proteasome, and the OTU-family DUB, OTUD4, has been shown to interact with both ALKB proteins. However, the DUB activity of OTUD4 is not required to stabilise ALKB2/3, suggesting it acts as a scaffold for other enzymes to prevent proteolysis [234,235]. USP7 and USP9X were further identified to be associated with OTUD4 and ALKB3, with overexpression of wildtype, but not mutant, USP7 or USP9X suppressing the ubiquitylation and subsequent degradation of ALKB3 in the presence of OTUD4 (Figure 3C). Levels of these oxidative demethylases are kept at low levels under normal conditions, with the response to DNA alkylation requiring a very rapid response and stabilisation by this DUB complex.

DUB regulatory pathways of Ub signalling in genome stability tend to vary widely, given the complexity of the Ub code and the number of DDR pathways and factors involved. However, there are common themes, with DUB activity able to stabilise proteins preventing proteasome-mediated degradation, altering of binding interfaces to recruit specific repair factors, and altering the localisation or activity of the protein(s) to ensure proficient repair. Other UBLs, such as SUMO, NEDD8, UFM1, and ISG15 [19,30], have been implicated in genome stability pathways and as such, deconjugating enzymes play a regulatory role in these processes. As with Ub, several UBLs are linked to the p53/MDM2 signalling axis, which is not described in detail here but reviewed elsewhere [19,236]. Instead, direct deUBLylation of DDR factors will be explored.

## ULPs in genome stability

### DeSUMOylation

SUMO has been established to have a critical regulatory role in the DDR. There are several SUMO paralogues within the human genome, with SUMO2 and SUMO3 sharing 97% sequence similarity, while SUMO1 shares only 47% similarity to SUMO2. SUMO4 and SUMO5/SUMO1P1 have also been reported [237]. Similar to Ub, SUMO1-3 can be conjugated to target substrates through an enzymatic cascade with an E1 (SAE1/2), E2 (Ubc9, also known as UBE2I) and several E3s (e.g. PIAS-family proteins), while removal is predominantly carried out by sentrin-specific proteases (SENPs) [238,239]. SUMO is highly conserved and similarly to Ub, is expressed as an immature precursor that is processed by SENP1 to enable conjugation to substrate lysine residues, with the possibility of polySUMO2/3 chains being formed, predominantly via K11. SENP1-3 and SENP5 are related to yeast Ulp1 in having preferred C-terminal hydrolase activity, with SENP6/7 related to Ulp2 and having preferred isopeptidase activity to clear longer SUMO2/3 chains. Other classes of SUMO proteases include DeSI-1/2 (DeSUMOylated Isopeptidase 1/2) and USPL1, with the latter localised to Cajal bodies and having isopeptidase activity.

Regarding the role of SUMOylation in genome stability, SUMO1 and SUMO2/3 can be detected in IR-induced foci (IRIF), laser-induced damage, DSBs, and can be precipitated from damaged chromatin, with SUMOylation known to play a role in HR and NHEJ pathways [158,240–243]. Several targets are known, such as MDC1 [244], RPA [242,245], FANCI-D2 [246,247], with the outcome of the target SUMOylation often to produce an altered recruitment platform, such as for SIM-containing proteins or STUbLs like RNF4, the latter highlighting the interplay between Ub and SUMO signalling in genome stability [248]. Indeed, activation of RNF4 through binding to polySUMO chains via its tandem SIMs can result in the formation of Ub-SUMO hybrids that is coupled to VCP/p97-mediated protein extraction from damaged chromatin and often resulting in proteasome-mediated degradation [249–251].

SUMO proteases tend to prevent the formation of extended chains and various SENP-family members have been identified to have key roles in DDR pathways. SUMO availability, through maturation or recycling of free SUMO from conjugates, enables proper HR and NHEJ signalling [242,252,253]. Loss or knockdown of SENP6 dramatically increases the level of long SUMO2/3-conjugates in the cell and enlarged SUMO foci at PML bodies, which can be rescued by supplying exogenous SUMO isoforms [254,255]. The non-redundancy, altered localisation, and preferences for SUMO protease activity mean it is not surprising that knockdown of individual SENP-family enzymes results in specific HR or NHEJ deficiencies [242,254,255].

#### SENP7 in DSB repair and chromatin relaxation

**Figure 4.**
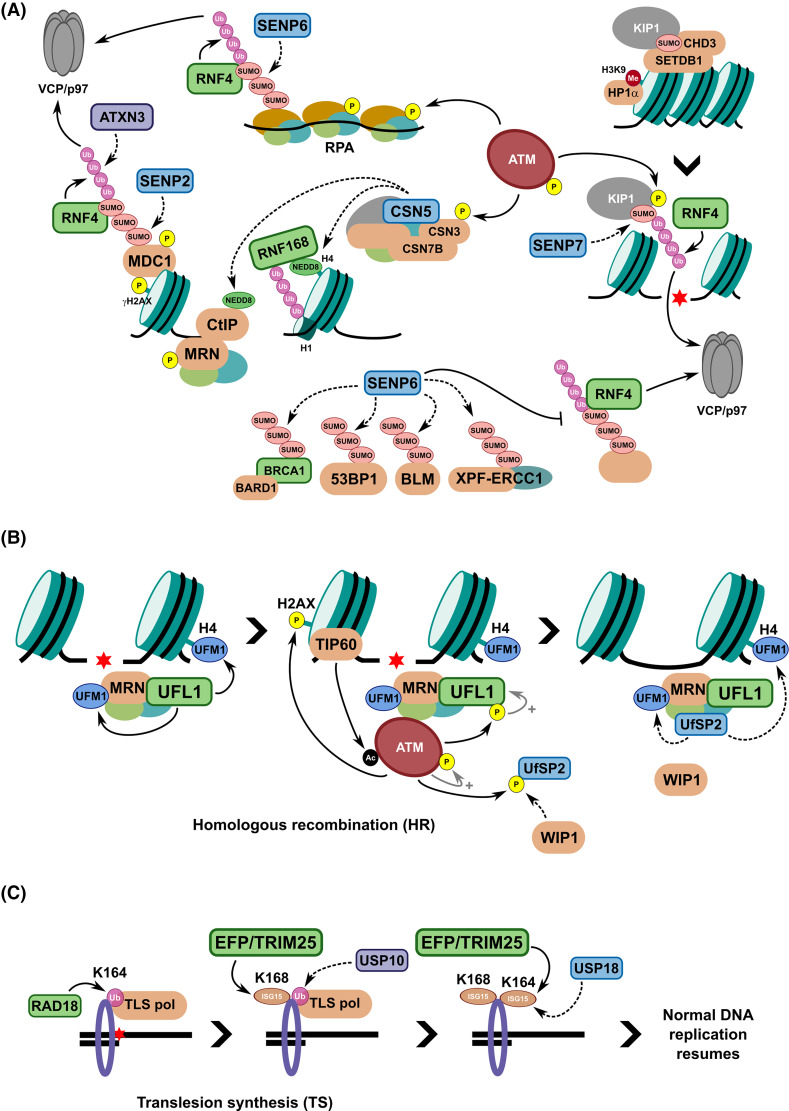
Overview of the role of ULPs in DDR signalling pathways. (**A**) The role of SENPs (SUMO ULPs) and the CSN (NEDD8 ULP) in DSB repair signalling. SENP6 is required to regulate SUMO2/3 levels on critical factors for DSB repair such as BRCA1-BARD1, 53BP1, BLM, XPF-ERCC1, and RPA to maintain active levels in an RNF4- and VCP/p97-dependent manner. SENP7 can deSUMOylate KAP1 to release its interaction with the chromatin remodeller, CHD3, which, along with phosphorylation by ATM, ensures chromatin relaxation in response to DSBs within heterochromatin regions. SENP2 and ATXN3 regulate the chromatin retention of MDC1 upon DNA damage in an RNF4- and VCP/p97-dependent manner, ensuring DDR signalling to repair the damage. The COP9 signalosome (CSN) is recruited to DSBs in a NEDD8-dependent manner, with CSN3 phosphorylated by ATM. CSN can remove NEDD8 modifications from Cullin-RING ligases (CRLs), histones, and other proteins during the DDR. (**B**) UFMylation of MRE11 and histone H4 in response to DNA damage occurs via the E3 ligase UFL1, which is recruited to DSBs through an interaction with the MRN complex at DSBs. H4 UFMylation enables downstream recruitment of the acetyltransferase TIP60 (via SUV39H1-mediated H3K9me3 formation), which acetylates ATM thereby promoting its activation and interaction with MRN. ATM kinase phosphorylation of itself and H2AX at S139 amplifies the DDR signalling for repair by HR. ATM-mediated phosphorylation of S462 on UFL1 further enhances UFL1 activity, resulting in a positive feedback loop. Recruitment of one of the two known UFM1 ULPs, UfSP2, to DSBs is prevented by inhibitory ATM-mediated phosphorylations at its S374/S381 residues, which are removed by the WIP1 phosphatase, allowing UfSP2 to bind MRN to deUFMylate H4 and dampen the ATM DDR signal upon successful repair. (**C**) During S phase, DNA damage blocking or stalling of the replication machinery leads to mono-ubiquitylation of PCNA at K164 by RAD6/RAD18, resulting in recruitment of more error-prone Y-family TLS polymerases and bypass of the lesion. To recycle unmodified PCNA and resume normal replicative processes, the E3 ligase EFP/TRIM25 can attach ISG15 to K168 on PCNA, leading to USP10-mediated removal of the mono-Ub on PCNA. EFP/TRIM25 can ISGylate K164, likely to prevent re-addition of Ub, before both ISG15 modifications are removed by USP18. How this mechanistically works in parallel with the established action of USP1-UAF1 is unknown. DUB/ULP activity is represented by dashed arrows.

One example is SENP7, which is required for HR-mediated repair of DSBs, with a role in chromatin relaxation to allow proficient DNA repair. KRAB-associated protein 1 (KAP1) is a heterochromatin protein that is SUMOylated in its C-terminal bromodomain (Figure 4A). Upon SUMOylation, KAP1 interacts with the chromatin remodeller CHD3 and the histone methyltransferase SETDB1 via their SIM domains [254]. CHD3 is a chromodomain-containing helicase that binds and distorts nucleosome DNA while SETDB1 is a H3K9 histone methyltransferase. Chromatin compaction by CHD3 and H3K9 trimethylation by SETDB1 results in heterochromatin formation and propagation via the heterochromatin protein, HP1α. Upon DNA damage, however, activated ATM releases the repressive KAP1-HP1α interaction by phosphorylation at S824 adjacent to the KAP1 bromodomain [256]. This damage-dependent phosphorylation also disrupts the SUMO-SIM interaction between SUMOylated KAP1 and CHD3, thereby promoting chromatin decompaction to allow efficient DNA repair. Depletion of SENP7, and therefore hyper-SUMOylated KAP1, fails to relax chromatin in response to damaging reagents due to increased chromatin retention of CHD3, preventing HR-mediated repair. SUMOylated KAP1 is also counteracted by the STUbL RNF4 via a concomitant interaction with the phosphorylated S824, with the resulting ubiquitylation leading to VCP/p97-mediated extraction and subsequent degradation of KAP1, providing a further mechanism for chromatin decompaction [257,258]. Therefore, while SUMO modifications can promote repressive chromatin sometimes advantageous in DDR-related transcriptional regulation, proficient repair in heterochromatin regions requires the removal of these marks either through STUbL-mediated degradation or via direct deSUMOylation.

#### SENP2 regulates MDC1 chromatin retention

While Ub signalling is widely known to regulate the decision between HR and NHEJ in repairing DSBs, the deSUMOylase SENP2 has also been identified as a key factor in these processes. As noted above, SENP-family proteases are required to maintain the supply of SUMO proteins available for conjugation to target proteins and thereby promote DDR signalling, such as to promote HR. Knockdown of SENP2 results in reduced RAD51 loading and reduced HR proficiency in cells in response to CPT and olaparib, while overexpression of SENP2 leads to increased NHEJ signalling due to the increased chromatin retention of MDC1, resulting in increased chromosome aberrations [255]. SENP2 is constitutively active and retained on chromatin to prevent excessive polySUMO2/3 chains forming on MDC1, thereby preventing engagement by the STUbL RNF4, which otherwise would extract MDC1 from chromatin and target it for degradation via VCP/p97. Loss of SENP2 leads to reduced chromatin bound MDC1, leading to increased sensitivity to IR or other DSB-inducing agents (Figure 4A). The VCP/p97-associated DUB, ATXN3, is also recruited to SUMO at sites of DSBs and is thought to counter RNF4-mediated poly-ubiquitylation to protect MDC1 [259] and RNF8 [260] (Figure 4A). Therefore, the levels of SENP2 need to be precisely regulated and it is worth noting that *SENP2* is one of several genes, along with *RNF168* and *USP13*, on the amplified region of chromosome 3q found in many aero-digestive tract cancers for example.

#### SENP6 enables controlled extraction of proteins from chromatin in the DDR

As an extension to the activity of SENP2 on MDC1, another deSUMOylase, SENP6, also regulates the chromatin retention of the FANCI-D2 heterodimer (Figure 3A). As described above, ATR-mediated phosphorylation and FA core complex-mediated ubiquitylation enables regulated recruitment of FANCI-D2 to sites of ICL damage. To enable proficient repair, the FANCI-D2 clamp needs to be removed or extracted from chromatin, and this is enabled through PIAS4-mediated polySUMO2/3 chain formation leading to RNF4 recruitment and subsequent extraction via VCP/p97 [246]. SENP6 depletion reduces the chromatin retention of FANCI-D2 and may lead to premature removal of FANCI-D2 via RNF4, reducing efficiency of ICL repair. Once again therefore, proper expression and recruitment of deSUMOylases is key to enable proficient DDR signalling.

More recently, the roles of SENP6 in genome stability, in addition to those described above for SUMO availability and FANCI-D2 chromatin retention, have been expanded [261]. Through screening of proteins modified with polySUMO2/3 chains upon SENP6 depletion, an array of DDR proteins, including BRCA1-BARD1, 53BP1, BLM, and XPF-ERCC1, were shown to be regulated through polySUMOylation and RNF4-mediated chromatin extraction (Figure 4A). SENP6 is proposed to maintain these proteins in a hypo-SUMOylated state in basal conditions and counteracts excessive polySUMO2/3 chain formation in response to HU-induced replication stress. Reduced SENP6 levels led to dysregulated recruitment or aberrant retention of proteins at damaged sites, increased SUMO2/3 levels at UV or IR-induced damage sites, and reduced DNA repair proficiency.

Generally, deSUMOylases seem to act as buffers or thermostats to maintain minimal SUMOylation of DDR-related proteins, enabling a rapid response to DNA damaging agents. Excessive SUMOylation can lead to premature removal of proteins from chromatin via RNF4 and VCP/p97, while excessive deSUMOylation can result in the aberrant retention of proteins at sites of DNA damage (e.g. MDC1) leading to more error-prone repair pathways. The role of SUMOylation in chromatin compaction and possibly in nuclear condensate formation [261] also suggests a more global method for regulating the availability of DDR proteins and access to sites of DNA damage to ensure efficient repair. The SENP-family of proteins seem to be central to regulate this, with their expression and constitutive activity crucial to maintain genome stability. Given the global role of SUMOylation in a multitude of signalling pathways, it would be difficult to design related inhibitors to specific SUMO proteases but their expression levels, either at the level of RNA or protein, or cellular localisation could be used as potential markers for disease-driving signalling pathways to explore. Protein SUMOylation tends to act in parallel to Ub-signalling in the context of genome stability, often via RNF4 and VCP/p97. However, for other UBLs, this is not always the case, meaning ULPs for these other modifications likely regulate the DDR via other mechanisms.

### DeUFMylation

The latest UBL to be identified is UFM1, an 85 amino acid long, 9.1 kDa protein [262]. As with other UBLs, UFM1 needs to be processed to a mature form by removing two C-terminal amino acids (S84 and C85) to expose a conserved glycine (G83 in humans) enabling conjugation to target proteins. UFM1 substrate conjugation occurs via a stepwise manner analogous to Ub and SUMO with an E1 (UBA5), E2 (UFC1) and E3 (UFL1 with cofactors UFBP1 and CDK5RAP3) enzymatic cascade, with polyUFM1 chains able to form via an internal lysine at amino acid position 69 [19,263–265]. Two UFM1-specific cysteine proteases have been identified, UfSP1 and UfSP2, to reverse UFM1 conjugation on target substrates, possibly to regulate UFC1 activity, and to process the expressed precursor form [266].

UFMylation has predominantly been linked to roles in endoplasmic reticulum (ER) homeostasis, fatty acid metabolism, autophagy, neurodevelopment, and liver development [263,264,267,268]. A major substrate for UFMylation is the 60S ribosomal subunit RPL26, which occurs on stalled ribosomes at the ER. Structural evidence suggests the role of UFM1 modification in this context is to release 60S ribosomes from the SEC61 translocon to regulate protein homeostasis at the ER [269,270]. UFM1 substrates have additionally been identified that have roles in DDR signalling and telomere maintenance pathways, including MRE11 [271] and histone H4 [272]. The UFM1-specific E3 ligase UFL1 is proposed to be recruited to IR-induced sites of DSBs, where it can interact with, and UFMylate MRE11 at K281 and K282. MRE11 UFMylation is thought to stabilise the MRN complex at sites of damage to activate the ATM kinase, while recruited UFL1 can also UFMylate histone H4 at K31, aiding to localise TIP60 to DSBs to further increase ATM activity. A positive feedback loop to amplify the ATM activation signal is thought to exist, with ATM-mediated S462 phosphorylation on UFL1 elevating its E3 activity [272] (Figure 4B). Furthermore, MRE11 mutants at target UFMylation sites abrogated its interaction with telomere repeat-binding protein, TRF2, resulting in reduced telomere length in HeLa cells and haematopoietic zebrafish cells [273]. While no DSB repair defects linked to ATM activation and DSBs were detected in this case, it is possible differences in cell lines used and variable context requirements exist for UFMylation in DDR pathways, although further investigation would be required.

#### UfSP2 regulation of H4 UFMylation

As opposed to other UBLs that are conjugated to substrates via a C-terminal di-glycine motif, UFM1 exhibits a C-terminal VG, masking it from other DUBs and ULPs ensuring specificity and fidelity in UFM1 signalling pathways. UfSP1 and UfSP2 share close structural homology within their catalytic domains, but several regulatory loops distinguish their proteolytic activities, suggesting they are regulated via allosteric means. Furthermore, an N-terminal extension in UfSP2 localises it to the ER membrane via ODR4 while UfSP1 is predominantly cytosolic, likely establishing alternative substrates, with UfSP1 important for UFM1 maturation and removing the auto-inhibitory UFC1 UFMylation, and UfSP2 for regulating UFMylated ribosomes [265]. Given the sub-cellular localisation of UfSPs, their roles in genome stability are unclear. A recent study, from the same group that identified H4 UFMylation, proposed a role for UfSP2 in modulating ATM activation upon IR-induced DSBs [274]. Like UFL1, recruitment was via the MRN complex but occurred at later time points following DNA damage due to an ATM-dependent phosphorylation on UfSP2 at S374 and S381 reducing the binding to MRE11. However, this could be reversed via the phosphatase WIP1, which enabled UfSP2 recruitment, H4 deUFMylation, and dampening of the ATM activation loop (Figure 4B). The mechanism of how UfSP2 is shuttled to DSB foci in the nucleus, and the kinetics between UFL1 and UfSP2 localisation and activity, will need further exploration.

### DeISGylation

ISG15 is a 15 kDa protein that contains two UBL domains separated by a flexible linker. Like UFM1, ISG15 is expressed as a 165 amino acid, 17 kDa precursor, which is processed to the mature form through removal of the C-terminal 8 amino acids to expose a diglycine motif available for substrate conjugation. Modification of substrate proteins at target lysine residues with ISG15 is through an enzymatic cascade via an E1 (UBA7/UbE1L), E2 (UBE2L6/UBCH8), and one of three E3 ligases (HERC5, HHARI, EFP/TRIM25). USP18 [275,276], along with USP5, USP14, USP21 and recently USP16 and USP36, have been identified to have deISGylase activity to reverse the modification [277]. Critically, expression of *ISG15* and the ISGylation machinery is stimulated by type I interferons, cytoplasmic nucleic acid detection pathways, and other cellular stresses, with ISG15 predominantly known for its role in the classical innate immune response during viral infections to limit viral replication [30,278,279]. ISG15 has more recently been identified by several groups as acting in response to replication-coupled DNA damage, with *ISG15* expression and substrate ISGylation detected in response to UV light in certain cell types [280]. In addition, replication stress leading to the accumulation of cytoplasmic DNA has been proposed to activate innate immune signalling via the cGAS-STING-Tbk1 pathway, resulting in *ISG15* expression and target protein ISGylation, including of DNA replication-associated proteins, preventing replication fork stalling and genome instability [281].

#### USP18 ensures high-fidelity replication resumes following TLS

In the DDR, ISG15 modifications have been identified to overlap with Ub-related signalling nodes for replication-coupled DDR pathways, possibly linked to observations for induced expression of the ISGylation machinery as a result of replication stress [19,30,281,282]. TLS facilitates replication past DNA lesions induced by UV light, for example, with a key event being PCNA mono-ubiquitylation at K164 to promote displacement of replicating polymerases with lower-fidelity polymerases. The ISG15 E3 ligase, EFP/TRIM25, was proposed to bind mono-ubiquitylated PCNA leading to ISGylation at K168, recruitment of USP10 to remove the Ub mark, and causing the release of TLS polymerases and other factors, such as REV1, from PCNA to terminate error-prone replication [280]. ISGylation at K164 likely prevents subsequent TLS initiation before USP18 removes the ISG15 marks to enable reloading of replicative polymerases and resumption of high-fidelity DNA replication. USP18-mediated removal of the ISG15 mark is a key step following damage and ISGylation is critical for preventing excessive UV-mediated mutagenesis and genome instability [280,283] (Figure 4C). How this ISG15- and USP10-mediated signalling functions in parallel to the more established USP1-UAF1 mediated regulation of PCNA mono-ubiquitylation will need further exploration.

#### DeISGylases may regulate K63-linked polyUb formation in DDR signalling

ISG15 can form mixed chains with Ub through conjugation onto K29 of Ub, indicating a directly overlapping role in Ub signalling [284], while ISGylation of Ubc13/UBE2N may also regulate the DDR. Ubc13 is an E2 conjugating enzyme important for several DDR signalling pathways by generating K63-linked polyUb chains as recruitment platforms for multiple DSB repair factors [285]. ISGylation at K92 of Ubc13 inhibits its activity by disrupting its ability to form a thioester bond to Ub [286]. No direct link to the DDR has been identified for proper removal of ISG15 from Ubc13 as K63-linked polyUb is required for numerous cell signalling pathways but given the intersection between the immune response and DDR signalling, it would be intriguing to see how deISGylation may regulate these processes.

USP18 is predominantly the deISGylase thought to act in cells particularly as its expression aligns with the interferon response and *ISG15*. Other USP-family members such as USP5, USP14, USP16, USP21, and USP36 [277,287], have all shown deISGylase activity *in vitro* on top of their primary protease activity against other UBLs and/or Ub. Whether these moonlighting activities are relevant to ISG15 biology remains to be seen, but the development of specific tools to probe activity in response to DNA damage will likely aid future work.

### DeNEDDylation

NEDD8 is a UBL expressed as an 81 amino acid precursor before processing by DEN1 (also known as NEDP1 or SENP8) to remove the last 5 amino acids to produce the mature form. NEDDylation is most commonly known to activate Cullin-RING ligases (CRLs) by modification of the core Cullin subunit to enhance their E3 Ub ligase activities, reviewed in more detail elsewhere [288]. NEDD8 has a single E1 (NAE1) and two E2 conjugating enzymes (UBC12, also known as UBE2M, and UBE2F) to transfer to the target Cullin subunit [289]. With respect to genome stability, NEDD8 conjugates are enriched at sites of DNA damage, with RNF111-mediated NEDDylation of H4, to promote RNF168 recruitment, and CtIP regulating the response to DSBs [23,34,290–294]. The COP9 signalosome (CSN) consists of 8 subunits required for the removal of NEDD8 from CRLs, resulting in reduced E3 ligase and auto-ubiquitylation activity. CSN5 is the active JAMM-type metalloprotease within this complex. CSN3 is a substrate for the ATM kinase and the CSN has roles in NER and protection against DSBs, with CSN recruited to DSBs in a NEDD8-dependent manner, indicating a key role in genome stability signalling pathways [293] (Figure 4A). Furthermore, CSN7B can be recruited to sites of DNA damage caused by MMC or IR to remove NEDD8 from CRLs, and possibly other E3 Ub ligases or histone proteins. Knocking out CSN7B, as well as the use of NEDD8-relevant inhibitors such as MLN4924 (NAE1 inhibitor) and CSN5i-3 [295], reduced ATM-dependent signalling and apoptosis, while shifting repair preference towards alt-NHEJ via PARP1 over HR, so altering genome stability pathway choice in response to DSBs [296]. Alt-NHEJ is an error-prone backup repair pathway that relies on PARP1 recognition of the DSB to recruit the MRN complex and CtIP, resulting in short-range resection of the DNA (2–20 nucleotides), filling in by DNA polymerase Q, and sealing of the nick by XRCC1 and DNA Ligase III [297,298].

## Conclusions and future perspectives

### Redundancy in DUBs/ULPs

The identification of many DUBs, plus additional ULPs, suggests that proper regulation of Ub and UBL modifications is critical to the DDR. Several DUBs, such as USP7, USP1, BAP1, appear to play distinct roles in multiple repair pathways, while critical Ub/UBL signalling nodes, such as modified PCNA and H2A, are targets for multiple DUBs/ULPs. The latter evidence of functional redundancy likely provides a safety net for genome stability, with backup systems to ensure damage is repaired. Another possibility is that multiple DUBs act in temporally or spatially different contexts for genome stability, depending on other signalling cues or the type of damage. For example, different DUBs may act during G0 or G1 versus S/G2 phase of the cell cycle (e.g. USP11), or as noted for the KAP1-CHD3 interaction and relevant to INO80, the chromatin architecture or local environment is likely important to dictate the Ub/UBL signalling events to ensure efficient repair processes.

The complexity of the Ub/UBL code and the likely presence of mixed or branched Ub/UBL chains [299–302] further lends credence that multiple DUBs/ULPs, with different enzyme kinetics or substrate specificities, are required to ensure regulated signalling for repairing DNA damage and to prevent unnecessary spreading of repair processes (e.g. hyper-resection). POH1 and USP5 possibly acting in concert is one example, while the K63-specific activity of ZUP1 may potentially allow Ub chain editing by neighbouring E3 ligases and/or regulated extraction of target proteins from chromatin via VCP/p97. Relevant for VCP/p97 is the combined activity of DUB and SENP partners to regulate the STUbL, RNF4, required to target proteins for degradation. More recent evidence for ISG15 and UFM1 modifications overlapping with that of Ub signalling, such as on PCNA or the MRN complex, lends further speculation that altered activities of DUBs and deUBLylases need tight regulation to ensure co-ordinated removal of the modifications. Additional enzyme activities for non-protein [15] or non-lysine [16] ubiquitylation may also need to be considered.

Some DUBs, such as OTUD4 and OTUB1, promote efficient DDR signalling through non-catalytic roles, either as allosteric regulators of components or as scaffolds to recruit other DUBs to sites of damage. Indeed, large multi-subunit DUB/ULP complexes, such as BRCA1-A, PR-DUB, and CSN, are likely regulated on multiple levels with respect to cellular localisation, substrate specificity, and allosteric regulation. Recent developments in single particle cryo-EM alongside high-quality sample preparation and biophysical techniques [303] allows specific questions about these molecular machines to be answered to clarify their role in cell signalling, such as for genome stability. Preparing uniform and designer substrates may also enable any questions about DUB/ULP target specificity to be addressed, which has helped disentangle some of the apparent DUB redundancy observed for H2A ubiquitylation in DSB repair for example [122].

### Regulating the regulators

Many DUBs and ULPs tested *in vitro* can show varying degrees of activity or specificity in their ability to remove Ub/UBL marks. How they are localised to sites of DNA damage and how they engage with specific substrates is more difficult to ascertain, as few DUBs/ULPs contain cellular localisation signals or substrate binding domains. As noted above, some DUBs exist as larger multi-subunit complexes to ensure correct recruitment and activity, such as BRCC36 that is recruited to DSBs via RAP80. Other DUBs likely have interacting partners such as ZUP1, with several UBDs to restrict its activity to K63-linked polyUb chains, interacting with the RPA complex to localise it to sites of ssDNA. Some DUBs are directly modified, either with Ub/UBLs or via DDR-mediated phosphorylation by ATM/ATR/DNA-PKcs, to enable recruitment or shuttling to sites of damage, such as for VCPIP1 and BAP1. ATR-mediated phosphorylation of the USP1-UAF1 complex and of its substrate, ubiquitylated FANCI-D2, has also been shown to regulate the formation of the enzyme-substrate complex for efficient Ub removal.

Related to USP1-UAF1, is how some DUBs function in multiple different DDR signalling pathways. USP1 can be regulated through auto-cleavage and via its UAF1 cofactor, which itself is a cofactor for other DUBs USP12 and USP46 not involved in genome stability signalling [166]. USP7 is another DUB implicated in various pathways of DNA damage signalling, including for p53-MDM2, HR, NER, alkylation repair, plus others, with scaffolding or interacting partners helping to recruit its activity in some cases.

### Technology development for DUB/ULP investigation

Utilisation of genetic screens, such as siRNA or CRISPR-Cas9, has identified many of the DUBs and ULPs described here, with improvements in technology enabling genetic and chemo-genetic interactions to be discovered [304–308]. CRISPR screens have helped overcome some of the potential off-target effects of RNAi screens, which has identified or confirmed major signalling nodes within DDR signalling, while subtle regulators of the pathways, such as E3 ligases or DUBs/ULPs, either show some redundancy or have cell-type specific effects [303,307]. Expanding on these monogenic screens to identify synthetic lethal genetic interactions, such as for USP1 and BRCA1 [167], may expand the repertoire of potentially druggable targets in particular genetic backgrounds, whether through mono- or combination therapy. Alongside the discovery of the factors involved, increasing knowledge of the mechanisms by which they act in the DDR, and how they remove the modification from substrates, has been enabled by recent advances in technology development [303]. Progress in mass spectrometry techniques and proteomics analysis, alongside diglycine-enrichment and other tools, has allowed detection of the Ub/UBL linkages relevant to DNA damage signalling. Coupling this to semi-synthetic and designer approaches for producing Ub/UBL substrates, such as for UBLs [277] and mixed/branched chains [309,310], has helped to clarify the activity and specificity of DUBs and ULPs. Further enhancement in structure prediction using AlphaFold [311–314] and the rapid progress in structural biology using cryo-EM alongside complementary techniques, has provided hitherto unmatched detail regarding the regulation and activity of DUBs/ULPs.

### DUB/ULP targets in drug discovery

Selective inhibition of DUBs/ULPs is an active avenue for investigation regarding anti-cancer therapeutics. The involvement of USP1 and USP7 in multiple pathways allows specific targeting of these DUBs to sensitise cells to chemotherapy due to the dysregulation of multiple parallel DDR signalling pathways. For example, ML323 is a selective USP1-UAF1 inhibitor that has shown promise to inhibit FA and TLS signalling to maximise cancer cell cytotoxicity with few off-target effects [315]. Recent structural characterisation of inhibitor binding will further progress drug development in this case, with additional drugs currently in Phase 1 clinical trials such as KSQ-4279 [53]. Indeed, the increased mechanistic and structural characterisation of DUBs/ULPs with their targets or inhibitors, combined with deep sequencing of patient samples, either at the genomic or expression level, will likely enable more precise patient stratification with reduced likelihood of developing resistance. Current efforts to inhibit DUBs/ULPs are reviewed elsewhere in more detail [53,142,316–318]. Overall, an enhanced understanding of Ub/UBL signalling via DUBs and ULPs in DDR signalling will provide a stable grounding to develop targeted strategies for treating human diseases, such as cancers.

## Data Availability

Data availability is not applicable to this article as no new data were created or analysed in this study.

## Open Access

Open access for this article was enabled by the participation of University of Manchester in an all-inclusive *Read & Publish* agreement with Portland Press and the Biochemical Society under a transformative agreement with JISC.

## Abbreviations

AP: abasic site
ATG: autophagy-related protein
ATM: ataxia-telangiectasia mutated
ATR: ataxia telangiectasia and Rad3-related protein
BAP1: BRCA1-associated protein 1
BER: base excision repair
CMG: CDC45-MCM-GINS
CPT: camptothecin
CSN: COP9 signalosome
DDR: DNA damage response
DeSI: deSUMOylating isopeptidase
DPC: DNA-protein cross-link
DSB: double-strand break
dsDNA: double-stranded DNA
DUB: deubiquitylase
FA: Fanconi anaemia
FAT10: human leukocyte antigen F locus adjacent transcription 10
FUBI: Finkel-Biskis-Reilly murine sarcoma virus (FBR-MuSV) ubiquitously expressed
GG-NER: global genome nucleotide excision repair
HR: homologous recombination
HU: hydroxyurea
ICL: interstrand cross-link
IR: ionising radiation
IRIF: ionising radiation induced foci
ISG15: interferon-stimulated gene 15
JAMM: JAB1/MPN/Mov34
KAP1: KRAB-associated protein 1
MINDY: motif interacting with ubiquitin (MIU)-containing novel DUB family
MJD: Machado-Joseph domain-containing protein
MMC: mitomycin C
MRN: MRE11-RAD50-NBS1
NEDD8: neural precursor cell expressed and developmentally down-regulated 8
NER: nucleotide excision repair
NHEJ: non-homologous end-joining
OTU: otubain domain-containing protease
PCNA: proliferating cell nuclear antigen
PIKK: phosphoinositide 3-kinase-like kinase
PIP: PCNA interacting peptide
PR-DUB: polycomb-repressive DUB
PTM: post-translational modification
RPA: replication protein A
SENP: sentrin-specific protease
SIM: SUMO-interacting motif
SSA: single-strand annealing
ssDNA: single-stranded DNA
STUbL: SUMO-targeted ubiquitin ligase
SUMO: small ubiquitin-like modifier
TC-NER: transcription-coupled nucleotide excision repair
TLS: translesion synthesis
TS: template switching
UAF: USP1-associated factor 1
Ub: ubiquitin
UBD: ubiquitin binding domain
UBL: ubiquitin-like protein
UCH: ubiquitin C-terminal hydrolase
UFM1: ubiquitin-fold modifier 1
UIM: ubiquitin-interacting motif
ULP: ubiquitin-like protease
URM1: ubiquitin-related modifier 1
USP: ubiquitin-specific protease
UV: ultraviolet
VCP: valosin-containing protein
VCPIP1: valosin-containing protein p97/p47 complex-interacting protein 1
ZUP1: zinc finger-containing ubiquitin peptidase 1
